# The PHA Depolymerase Engineering Database: A systematic analysis tool for the diverse family of polyhydroxyalkanoate (PHA) depolymerases

**DOI:** 10.1186/1471-2105-10-89

**Published:** 2009-03-18

**Authors:** Michael Knoll, Thomas M Hamm, Florian Wagner, Virginia Martinez, Jürgen Pleiss

**Affiliations:** 1 Institute of Technical Biochemistry, University of Stuttgart, Allmandring 31, 70569 Stuttgart, Germany; 2 Department of Molecular Microbiology, Centro de Investigaciones Biologicas Ramiro de Maeztu, 9, 28040 Madrid, Spain

## Abstract

**Background:**

Polyhydroxyalkanoates (PHAs) can be degraded by many microorganisms using intra- or extracellular PHA depolymerases. PHA depolymerases are very diverse in sequence and substrate specificity, but share a common α/β-hydrolase fold and a catalytic triad, which is also found in other α/β-hydrolases.

**Results:**

The PHA Depolymerase Engineering Database (DED, ) has been established as a tool for systematic analysis of this enzyme family. The DED contains sequence entries of 587 PHA depolymerases, which were assigned to 8 superfamilies and 38 homologous families based on their sequence similarity. For each family, multiple sequence alignments and profile hidden Markov models are provided, and functionally relevant residues are annotated.

**Conclusion:**

The DED is a valuable tool which can be applied to identify new PHA depolymerase sequences from complete genomes *in silico*, to classify PHA depolymerases, to predict their biochemical properties, and to design enzyme variants with improved properties.

## Background

In the past decade, polyhydroxyalkanoates (PHAs) gained industrial interest as biodegradable substitutes for non-degradable plastics. While poly *(R)*-3-hydroxybutyric acid (PHB) is the most widely studied and the best characterized PHA, a wide variety of PHAs with differences in flexibility and thermostability have been described. Many bacteria accumulate PHAs as storage compounds of carbon and energy [1-3]. PHAs have been assigned to two classes, depending on the number of carbon atoms of the monomers: short chain length PHAs (PHA_SCL_) with 3 to 5 carbon atoms per monomer and medium chain length PHAs (PHA_MCL_) with 6 to 15 carbon atoms per monomer. PHAs are degraded by intracellular and extracellular PHA depolymerases. Intracellular PHA depolymerases hydrolyze an endogenous carbon reservoir, the native PHA granules, consisting of the polymer with a surface layer of proteins and phospholipids. Extracellular PHA depolymerases degrade denatured extracellular granules which are partially crystalline and are lacking a surface layer [4,5]. Thus, depending on their substrate and its physical state, PHA depolymerases are grouped generally into four families: PHA depolymerases degrading the native intracellular granules (nPHA_MCL _depolymerases and nPHA_SCL _depolymerases) and PHA depolymerases degrading the denatured extracellular PHA granules (dPHA_MCL _depolymerases and dPHA_SCL _depolymerases). One exception of this classification is an extracellular nPHA_SCL _depolymerase from *Paucimonas lemoignei *which is active only against native PHA granules [6]. Additionally periplasmatic PHA depolymerases exist, as a PHA depolymerase from *Rhodospirillum rubrum *has been described recently to be located in the periplasm [7].

PHA depolymerases are carboxylesterases and belong to the α/β-hydrolase fold family [5].

As member of the α/β-hydrolase fold, two families including PHA depolymerases have also been described in the Pfam proteins families database [8]: the family of *Esterase PHB depolymerases *(Pfam accession code: PF10503) and the family describing the C-terminus of bacterial PHB depolymerases (Pfam accession code: PF06850).

With exception of a few intracellular nPHA_SCL _depolymerases, all PHA depolymerases have a catalytic triad (serine – histidine – aspartic acid) as active site. The catalytic serine is embedded in a GxSxG sequence motif (known as 'lipase box') as found in other α/β-hydrolases. Additionally, a conserved non-catalytic histidine near the oxyanion hole is found analogous to lipases [5,9]. The best studied PHA depolymerases are dPHA_SCL _depolymerases. They share a common domain architecture consisting of a short signal peptide, a catalytic domain (including the lipase box and the oxyanion hole), a short linker domain, and a substrate binding domain [10]. Depending on the location of the lipase box on sequence level relative to the oxyanion hole, two types of catalytic domains are known. Within the sequences of type 1 catalytic domains, the oxyanion hole can be found N-terminal to the lipase box, similar to lipases. Within the sequences of type 2 catalytic domains, the oxyanion hole is found C-terminal to the catalytic triad. In contrast to dPHA_SCL _depolymerases, dPHA_MCL _depolymerases possess no substrate binding domain. In these enzymes, the N-terminal region of the catalytic domain is assumed to function as substrate binding site [5].

The PHA depolymerase from *Rhodospirillum rubrum *which is described to be located in the periplasm [7] is a special case, as it has a catalytic domain similar to extracellular PHA depolymerases with a catalytic domain type 2.

For intracellular nPHA depolymerases no particular substrate binding domain has been described so far. A few intracellular nPHA_SCL _depolymerases have no lipase box, but have a catalytic triad consisting of cysteine, histidine, and aspartic acid. One member of this family is the nPHA_SCL _depolymerase of *Ralstonia eutropha *[11].

Only about 30 PHA depolymerases with experimentally validated PHA depolymerase activity have been described so far. The factors which mediate the capability of depolymerases to degrade PHAs with high specificity are not yet understood. Although the sequence similarity of PHA depolymerases to other known α/β-hydrolases like lipases and esterases is low and substrate specificity differs considerably, they belong to the same fold family and possess a highly conserved active site. From a systematic comparison of the PHA depolymerase family to other α/β-hydrolases, depolymerase-specific motifs can be derived. However, a data resource is still lacking which integrates sequence and structure information and provides tools for a systematic analysis of the sequence-structure-function relationship of PHA depolymerases. Therefore, the PHA Depolymerase Engineering Database (DED, ) has been designed to assist a comprehensive analysis of sequences, the annotation of new sequences and the design of mutants. For the analysis of lipases and esterases, the Lipase Engineering Database (LED, ) has previously been established and applied [12,13]. Comparison of the rules derived from the LED to the DED will help to understand differences of PHA depolymerases and other α/β-hydrolases, and will relate experimentally observed properties of PHA depolymerases to their sequence.

## Construction and content

To establish the PHA Depolymerase Engineering Database, the data warehouse system DWARF [14] has been applied. The DWARF system provides an automated retrieval tool to extract information on sequence, structure, or function from different source databases into a local data warehouse system. As a first step, 28 seed sequences of proteins with experimentally validated depolymerase activity (Table 1[15-33]) were stored in the database and annotated. These seed sequences were assigned to 6 previously described superfamilies based on their function [34]. Additionally the families of intracellular nPHA_SCL _depolymerases (lipase box), of which one family member has recently been described [35], and the family of periplasmatic PHA depolymerases including the PHA depolymerase from *Rhodospirillum rubrum *[7] were introduced. Thus, a total of 8 superfamilies were introduced:

**Table 1:** Experimentally validated PHA depolymerases, which were used as seed sequences to set up the DED.

**Accession number (gi)**	**Organism**	**Family**	**Reference**
3641686	*Ralstonia eutropha *H16	Intracellular nPHA_SCL _depolymerases (no lipase box)	[28]

75763431	*Bacillus thuringiensis *serovar israelensis ATCC 35646	Intracellular nPHA_SCL _depolymerases (lipase box)	[35]

22035160	*Rhodospirillum rubrum*	Periplasmatic PHA depolymerases	[7]

130002	*Pseudomonas oleovorans*	Intracellular nPHA_MCL _depolymerases	[18]
21689574	*Pseudomonas putida*		[17]

130019	*Ralstonia pickettii*	Extracellular dPHA_SCL _depolymerises (catalytic domain type 1)	[29]
1777951	*Alcaligenes faecalis*		[23]
116744367	*Bacillus megaterium*		[48]
1730532	*Paucimonas lemoignei*		[20]
7385117	*Paucimonas lemoignei*		[31]
1657610	*Paucimonas lemoignei*		[15]
1621355	*Paucimonas lemoignei*		[15]
531464	*Paucimonas lemoignei*		[45]
531466	*Paucimonas lemoignei*		[45]
75538924	*Pseudomonas stutzeri*		[26]
5360565	*Ralstonia pickettii*		
1381030	*Ralstonia pickettii*		

4033618	*Acidovorax *sp. TP4	Extracellular dPHA_SCL _depolymerises (catalytic domain type 2)	[25]
7209864	*Caldimonas manganoxidans*		[33]
565666	*Comamonas *sp.		[19]
75340123	*Delftia acidovorans*		[21]
47078657	*Schlegelella *sp. KB1a		[27]
1389770	*Streptomyces exfoliatus*		[24]
88192747	*Penicillium funiculosum*		[16]

15788987	*Paucimonas lemoignei*	Extracellular nPHA_SCL _depolymerases	[6]

34452163	*Pseudomonas alcaligenes*	Extracellular dPHA_MCL _depolymerases	[22]
29470160	*Pseudomonas alcaligenes*		[22]
21542177	*Pseudomonas fluorescens*		[30]


▪ intracellular nPHA_SCL _depolymerases (no lipase box)

▪ intracellular nPHA_SCL _depolymerases (lipase box)

▪ intracellular nPHA_MCL _depolymerases

▪ periplasmatic PHA depolymerases

▪ extracellular dPHA_SCL _depolymerases (catalytic domain type 1)

▪ extracellular dPHA_SCL _depolymerases (catalytic domain type 2)

▪ extracellular nPHA_SCL _depolymerases

▪ extracellular dPHA_MCL _depolymerases

**Figure 1:**
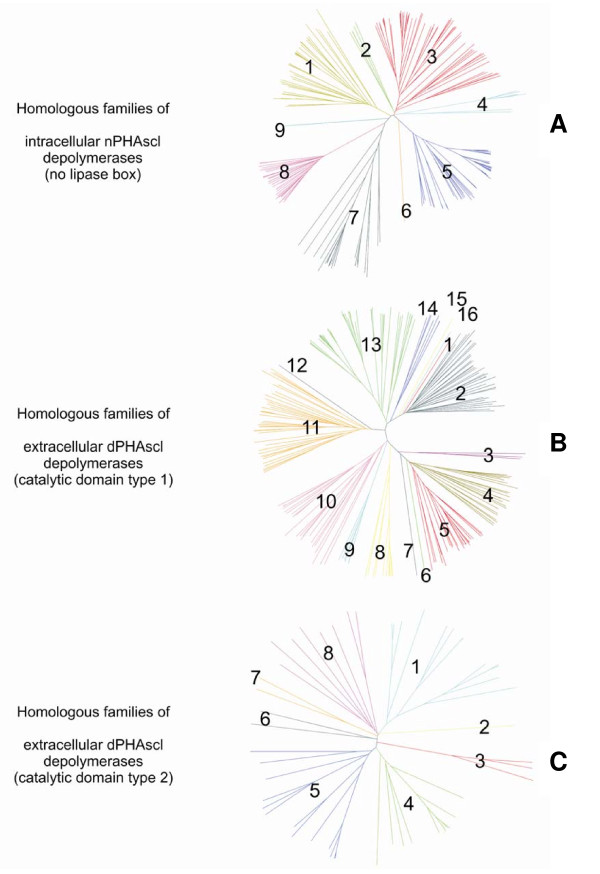
**Assignment of homologous families**. Homologous families of intracellular nPHA_SCL _depolymerases (no lipase box) [A], extracellular dPHA_SCL _depolymerases (catalytic domain type 1) [B], and extracellular dPHA_SCL _depolymerases (catalytic domain type 2) [C]. The homologous family number is indicated.

The DWARF system was further applied to populate the database with sequences obtained from a pool of selected sequences, which have been annotated as "depolymerase" in the GenBank [36]. This was done by performing a BLAST search [37] of each seed sequence in the database against the selected sequence pool derived from the GenBank. As a final population step, BLAST searches against the non-redundant sequence database at NCBI  were performed for each sequence with an E-value cut-off of E = 10^-50 ^to populate the database with more sequences. Superfamilies were subdivided into homologous families, which were introduced based on sequence similarity and phylogenetic analysis (Fig. 1). New protein entries were assigned to homologous families and superfamilies by their sequence similarity.

Sequence entries with more than 98% sequence identity, which originate from the same source organism, were assigned to a single protein entry. In case of multiple sequence entries for one protein, the longest sequence was set as reference sequence. For protein entries with available structure information, structural monomers were downloaded from the Protein Data Bank [38] and stored as structure entries. Secondary structure information was calculated applying the program DSSP [39] and displayed in the annotated multiple sequence alignments which are generated using ClustalW (v1.83) with default parameters [40]. Annotation information on structurally or functionally relevant residues (active site, disulfide bridges, signal peptide) was extracted from the NCBI entries and annotated in the DED. Information on experimentally validated depolymerases was manually added. Residues of the lipase box and the catalytic triad were manually annotated, which enables an easy identification of these residues for almost all PHA depolymerases based on multiple sequence alignments.

The PHA Depolymerase Engineering Database consists of 735 sequence entries which code for 587 different proteins. The proteins have been assigned to 8 superfamilies and 38 homologous families. The largest PHA depolymerase families are the intracellular nPHA_SCL _depolymerases (no lipase box) and the extracellular dPHA_SCL _depolymerases (catalytic domain type 1) with 224 and 234 protein entries, respectively, and account for 38% and 39% of all protein entries. Only one member of the family of periplasmatic PHA depolymerases was found, the PHA depolymerase of *Rhodospirillum rubrum*. For the families of extracellular dPHA_SCL _depolymerases (catalytic domain type 2) and the family of extracellular nPHA_SCL _depolymerases, structure information is available. Interestingly, two proteins from *Cupriavidus taiwanensis *and *Ralstonia eutropha *H16 which are annotated as "intracellular PHA depolymerase" in the GenBank were assigned to the family of extracellular dPHA_SCL _depolymerases (catalytic domain type 1) due to their sequence similarity (gi: 194292521, gi:74267419 [41]). The latter is reported to be highly active against artificial amorphous PHB granules, and is lacking a signal peptide, a linker domain, and a substrate binding domain. Another exception is the PHA depolymerase from *Pseudomonas sp. *which is annotated as "extracellular PHA depolymerase" in the GenBank but was assigned to the family of intracellular nPHA_MCL _depolymerases in the DED (gi:34452171).

## Utility and discussion

The DWARF system is an integrative bioinformatics tool to build up protein family databases into a local data warehouse system. The DWARF system has previously been successfully applied to build up the Lipase Engineering Database [12,13], the Cytochrome P450 Engineering Database [42], and the Medium-Chain Dehydrogenase/Reductase Engineering Database [43]. A local data warehouse has the advantage of a common and consistent data structure which enables systematic analysis of complete protein families. The DED is the first data source that integrates information on sequence, structure, and function of PHA depolymerases in a systematic and consistent format.

### Web accessibility

The database can be browsed on the level of sequence, structure, or organism. All protein entries are linked to the respective NCBI entries. Annotated multiple sequence alignments and phylogenetic trees that are visualized applying the program PHYLODENDRON  are provided via the online accessible version of the DED at . For each family, information of amino acid conservation is given as calculated by PLOTCON [44]. For each homologous family and superfamily, family-specific profile hidden Markov models were calculated by the HMMER program  to assist the classification of new PHA depolymerase sequences and the identification of new PHA depolymerase sequences from complete genomes *in silico*. A local BLAST interface is available to perform a BLAST search against the DED. A new dynamic user interface was developed which enables fast and easy integration of updated versions of the DED. The DED will be regularly updated by an automated script. For new sequence entries referring to a new structure in the Protein Data Bank (PDB), structure information is updated as well. New sequence and structure entries are classified into the homologous families and superfamilies based on their sequence identity.

### Analysis

**Figure 2:**
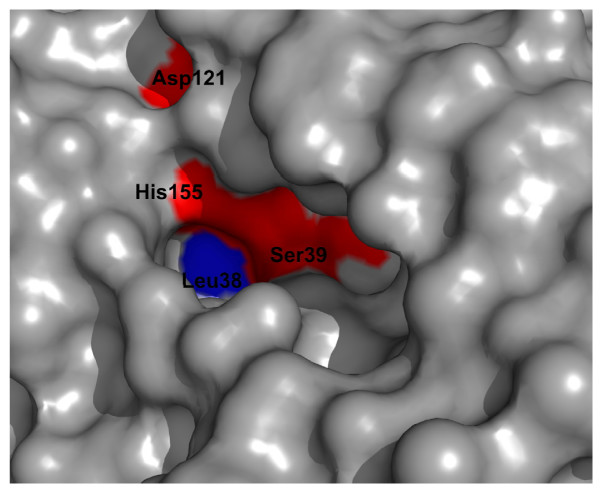
**Top view of the binding site of the PHB Depolymerase from *Penicillium funiculosum *(PDB entry **2D80, [46]). The catalytic residues are marked in red, the hydrophobic residue at position x_1 _of the Gx_1_Sx_2_G motif is marked in blue.

All PHA depolymerases in the DED possess a lipase box around the catalytic serine with a Gx_1_Sx_2_G sequence motif with the exception of the family of intracellular nPHA_SCL _depolymerases (no lipase box), which possess a catalytic cysteine instead of the lipase box. For particular PHA depolymerases it has been previously described that a hydrophobic residue is found at position x_1 _within the Gx_1_Sx_2_G motif [4,9,45]. This seems to be a common feature of almost all PHA depolymerases as seen from a systematic analysis of the DED family multiple sequence alignments. Thus, compared to other α/β-hydrolases like lipases and esterases, where a polar residue is most frequently found at position x_1_, this conserved residue of the Gx_1_Sx_2_G motif might be relevant to differentiate between lipases or esterases and PHA depolymerases on sequence level. This hydrophobic residue is solvent exposed and located near the catalytic serine at the bottom of a deep cleft, as seen in the structure of the PHB depolymerase from *Penicillium funiculosum *(PDB entry 2D80) [46] (Fig. 2). The hydrophobic residue at position x_1 _is tryptophan and isoleucine for the families of intracellular nPHA_SCL _depolymerases (lipase box) and periplasmatic PHA depolymerases, respectively. For the family of intracellular nPHA_MCL _depolymerases, the residue at position x_1 _is valine for almost all proteins. Although not possessing a lipase box, but utilizing a catalytic cysteine, all family members of the family of intracellular nPHA_SCL _depolymerases (no lipase box) also have a hydrophobic residue (almost all valine) at position cysteine-1. While the hydrophobic residue at position x_1 _differs among the families of intracellular PHA depolymerases, leucine and isoleucine are the most frequent residues at this position for extracellular PHA depolymerases. While all proteins of the family of extracellular dPHA_SCL _depolymerases (catalytic domain type 2) possess a hydrophobic residue at position x_1_, only 81% of the proteins of the family of extracellular dPHA_SCL _depolymerases (catalytic domain type 1) have a hydrophobic residue at position x_1_. All extracellular dPHA_MCL _depolymerases have an isoleucine at position x_1_. One exception is the family of extracellular nPHA_SCL _depolymerases, which neither possess a typical Gx_1_Sx_2_G motif nor has a hydrophobic residue a position x_1_. In this family, the Gx_1_Sx_2_G motif is altered to a AHSMG motif which can also be found in the family of Bacillus lipases (homologous family abH18.01 in the LED, ). One family member of this special family is the PHB depolymerase from *Paucimonas lemoignei*, for which also structure information is available (PDB entry: 2VTV) [6,47]. This PHB depolymerase has also special biochemical properties, as it is an extracellular nPHA_SCL _depolymerase degrading native granules, and is the only experimentally validated extracellular PHA_SCL _depolymerase not having a substrate binding domain. Within lipases and esterases, a polar residue is typically found at position x_1_. However, a few exception also exist among lipases and esterases, such as the of Candida antarctica lipase like family (homologous family abH37 in the LED) and the family of Bacillus carboxylesterases (abH11.1).

## Conclusion

The PHA Depolymerase Engineering Database (DED) has been designed to serve as a navigation and analysis tool of PHA depolymerases. It serves as a platform to analyze sequence-structure-function relationships and to classify new sequences by providing multiple sequence alignments, phylogenetic trees, and family-specific profiles. The DED hence provides a valuable source of information to investigate the family of PHA depolymerases in a systematic way, to identify new proteins from genomes, and to distinguish between PHA depolymerases and lipases. Thus, it paves the way for a deeper understanding of biochemical properties of PHA depolymerases and to design PHA depolymerases with improved properties.

## Availability and requirements

The PHA Depolymerase Engineering Database (DED) is online accessible at . All information on families of sequence and structure data, as well as alignments, phylogenetic trees, and family-specific profiles can be accessed by manual download.

## Authors' contributions

MK established and analyzed the database, and wrote the manuscript. TH carried out analysis and contributed to writing of the manuscript. FW programmed the dynamic user interface. VM contributed to establish the database. JP supervised the project and finalized the manuscript. All authors read and approved the final manuscript.
